# Latencies of conditioned vocal responses to hearing test tones in killer whales (*Orcinus orca*)

**DOI:** 10.3389/fnbeh.2024.1495579

**Published:** 2025-01-29

**Authors:** Jared Stephens, Alyssa W. Accomando, Kayla Nease, Brian K. Branstetter, Todd R. Robeck

**Affiliations:** ^1^National Marine Mammal Foundation, San Diego, CA, United States; ^2^Naval Information Warfare Center Pacific, San Diego, CA, United States; ^3^SeaWorld San Diego, San Diego, CA, United States; ^4^Naval Facilities Engineering Systems Command Pacific, Honolulu, HI, United States; ^5^SeaWorld Parks, Orlando, FL, United States

**Keywords:** marine mammal, reaction time, equal latency, loudness, sensation level

## Abstract

**Introduction:**

Perceived loudness is challenging to study in non-human animals. However, reaction time to an acoustic stimulus is a useful behavioral proxy for the assessment of perceived loudness. Understanding the effect of sound frequency and level on perceived loudness would improve prediction and modeling of anthropogenic noise impacts on marine mammals.

**Methods:**

In this study, behavioral hearing tests conducted with two killer whales were analyzed to capture conditioned vocal response latency, which is the time between the onset of the acoustic signal and the onset of the response (i.e., reaction time).

**Results:**

The results showed that vocal reaction times decreased with increasing sensation level (i.e., sound pressure level above the baseline hearing threshold), while the effect of frequency on reaction time varied between the subjects. Reaction time as a function of sound duration is described, and equal-latency contours are presented.

**Discussion:**

The data suggest that vocal reaction time decreases with increasing sensation level, therefore supporting the use of reaction time as a proxy for loudness perception in killer whales.

## 1 Introduction

Killer whales (*Orcinus orca*) rely heavily on their auditory sense for critical life functions such as communication and echolocation. They live in highly social groups that inhabit marine regions across the globe (Baird, 2000; Hoelzel et al., 2002). As the largest delphinid odontocete, killer whales are the best hearing surrogate for larger toothed whales such as sperm whales (*Physeter macrocephalus*) and beaked whales (family *Ziphiidae*), which are more difficult to study. Killer whales also have more sensitive hearing at lower frequencies than other model odontocete species [e.g., bottlenose dolphins (*Tursiops truncatus*)] in correlation with their larger functional interaural distance (Branstetter et al., 2017; Heffner and Heffner, 2008, 2018). Sources of low frequency anthropogenic noise (e.g., boat engines) are common in many of the regions that killer whales inhabit (Holt, 2008; NOAA, Fisheries, 2022).

The broad geographic distribution of killer whales, along with their sensitive hearing, heightens the risk of negative impacts from exposure to anthropogenic noise (Erbe et al., 2018). Auditory masking, hearing loss, and behavioral reactions could occur in response to anthropogenic noise (Nowacek et al., 2007; Weilgart, 2007; Williams et al., 2014b; Erbe et al., 2016), which may result in loss of foraging opportunities (Holt et al., 2011) and diminished communication space (Holt et al., 2009; Houghton et al., 2015). Audiograms, which plot a listener’s hearing sensitivity as a function of frequency, have helped establish noise safety criteria for underwater anthropogenic sound (Southall et al., 2019). However, these standardized audiograms have limited applicability as they only measure the lowest audible sound pressure level (SPL) of the listeners over their frequency range of hearing with no established relationship to perceived loudness. Loudness growth patterns change with sound frequency and other characteristics, including contextual factors (Suzuki and Takeshima, 2004; Finneran and Schlundt, 2011; Wensveen et al., 2014; Mulsow et al., 2015). Measuring subjective loudness is ideal for developing predictions of the likelihood that anthropogenic noise will result in behavioral responses (Finneran and Schlundt, 2011; Suzuki and Takeshima, 2004).

Changes in loudness perception with certain sound characteristics such as frequency, onset, and duration are poorly understood in non-human animals—in part because they are more difficult to study than humans. Equal loudness can be measured using subjective loudness judgments (i.e., the louder of two choices is indicated by the listener, or one sound is adjusted to match the loudness of the other sound) to determine the SPL of tones that are perceived to be equally as loud as a function of the target sound. This is easily tested in human participants but is challenging in odontocetes (Finneran and Schlundt, 2011; Pfingst et al., 1975a,b). Reaction time (RT), the time between the onset of a stimulus and the initiation of a response, has been used as a simple alternative to approximate perceived loudness in humans (Humes and Ahlstrom, 1984; Wagner et al., 2004) and marine mammals such as the harbor porpoise (*Phocoena phocoena*) (Wensveen et al., 2014), bottlenose dolphin (Mulsow et al., 2015), harbor seal (*Phoca vitulina*) (Kastelein et al., 2011), and California sea lion (*Zalophus californianus*) (Mulsow et al., 2015). The results of those studies suggest that, in many cases, reaction time is a suitable proxy for direct loudness measurements.

Reaction time data as a function of sound frequency can be used to generate equal-latency contours, which are functions that describe the sound levels at which reaction times are equal across the frequencies tested. Equal-latency contours from the bottlenose dolphin, harbor porpoise, harbor seal, and California sea lion resemble the shape of the audiogram at near-threshold sound pressure levels (Mulsow et al., 2015; Wensveen et al., 2014; Kastelein et al., 2011). This supports the use of the audiogram in predicting loudness perception for near threshold SPLs in marine mammals.

In the present study, conditioned killer whale vocal responses to sounds of different durations, sensation levels and frequencies were measured, and reaction time was evaluated as a potential indicator of loudness perception. Vocal reaction time was expected to decrease as sensation level increased, and resulting equal latency contours at lower sensation levels were expected to resemble the audiogram (Stebbins, 1966; Green, 1975; Pfingst et al., 1975a,b; Moody et al., 1980; Ridgway and Carder, 2000; May et al., 2009; Mulsow et al., 2015; Wensveen et al., 2014; Kastelein et al., 2011).

## 2 Methods

The data for the present study was collected opportunistically from Branstetter et al. (2023) as a part of a psychoacoustic study where the objective was to measure behavioral hearing thresholds as a function of signal duration. This section includes both a summary of the methods from Branstetter et al. (2023) (Sections 2.1–2.3) and the data analysis new to the present study (Section 2.4). Below, the terms response latency and reaction time have been used interchangeably to refer to the same concept.

### 2.1 Subjects

Two adult male Type C (fish eating) killer whales (*Orcinus orca*) participated in the present study: Whale E (20 years old, 3,920 kg, 599 cm in length) and Whale C (31 years old, 4,218 kg, 607 cm in length). Both whales had good species representative hearing (Branstetter et al., 2017). The whales were housed in a 21,000 m^3^ complex of interconnected pools of natural processed salt water at SeaWorld San Diego. The temperature varied seasonally between 12 and 14°C. Whales were fed a diet consisting of frozen–thawed whole fish at approximately 2 to 3% of their body weight per day. All fish were graded for human consumption and diets were supplemented with Vita-Zu Marine Mammal tablets without Vitamin A (Mazuri, St. Louis, MO). Each whale had a predetermined daily diet customized based on caloric needs, weekly body weight measurements, age and other factors as determined by the veterinary staff. The full daily diet was fed each day with no modifications based on session performance. Standard husbandry practices were employed whereby staff interacted with and fed animals during a minimum of eight variable sessions (3–30 min in duration) throughout the day extending over a minimum of 9 h per day (starting between 07:00 and 09:00). The study followed a protocol approved by the SEAS Animal Research Use Committee as well as an Institutional Animal Care and Use Committee at the National Marine Mammal Foundation (San Diego, CA). The behavioral test employed in the present study did not feature punishments or food-deprivation of any kind, and relied on the voluntary participation of the subjects, which could withdraw from the test at any moment.

### 2.2 Apparatus, signal generation and calibration

**Figure 1:**
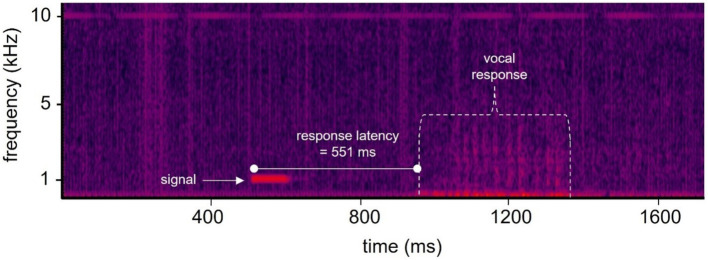
Spectrogram of a single trial showing the hearing test signal and vocal response. Each trial was recorded at a sample rate of 50 kHz. The onset of the stimulus relative to the start of the recording was always 500 ms. The vocal response latency in this example was 551 ms after the onset of the 100 ms duration signal, and 1,051 ms after the recording had begun. Color indicates relative acoustic intensity with brighter colors representing higher intensities.

Hearing tests were conducted in a medical pool (length 14.6 m, width 7.6 m, depth 2.7 m) with a hydraulic lifting floor located adjacent to two larger pools that were part of the killer whales’ habitat at SeaWorld San Diego. The pools were separated by large, underwater gates that allowed movement of water and conduction of sound but leaving the test animal temporarily physically isolated from conspecifics (Branstetter et al., 2017). The hearing test apparatus was an aluminum frame suspended underwater from a gate with a protruding station pad covered in closed-cell neoprene to prevent sound conduction (see Figure 1) from Branstetter et al. (2023). The point of interface between the gate and aluminum frame was also covered with closed cell neoprene to prevent sound conduction. The whales were stationed with their dorsum fully submerged while their rostrum touched the station pad during all hearing tests.

Hearing test signals were generated as described by Branstetter et al. (2023). Pure tones with durations between 0.1 ms and 2 s were digitally generated and projected by underwater piezoelectric transducers (see below). All tones had onset/offset ramps to reduce spectral splatter. The duration of the ramps was either 10 ms or 10% of the duration of the signal (whichever one was the shortest). All signals were attenuated (Tucker-Davis Technologies PA5 Programmable Attenuator, Alachua, FL) and amplified (Benchmark AHB2 Power Amplifier, Syracuse, NY) before being projected and calibrated using hearing test program (HTP) software (Finneran, 2003). Acoustic calibrations were conducted before sessions. Calibrations were conducted using a removable PVC apparatus that positioned the listening hydrophone where the whale’s lower jaw was located (57 cm behind the tip of the rostrum) when positioned at the station. Received signals were measured using either a TC-4033 or TC-4013 hydrophone (Teledyne Reson, Slangerup, Denmark) coupled to a voltage preamplifier (VP1000 or VP2000, Teledyne Reason, Slangerup, Denmark). The underwater sound projector was either suspended underwater from the back side of the gate [Lubell LL916 (Lubell Labs Inc., Whitehall, OH) for frequencies below 10 kHz] or attached to the aluminum frame [ITC1001 and ITC 1042 (International Transducer Corporation, Santa Barbara, CA) for 10–40 kHz or 80 and 100 kHz respectively].

### 2.3 Test sessions

On testing days (a maximum of 5 days per week), up to three hearing tests were conducted with each whale. Each hearing test typically lasted 5–10 min. Each hearing test consisted of approximately 5 to 10 “dives,” when the subject would submerge underwater and position at the station pad, during which 1 to 12 trials were conducted per dive. Trials were either signal trials where a sound was played or “catch” trials where no sound was played, with an equal probability of either trial type (trial order was determined by HTP software). A camera was attached to the PVC pipe on the aluminum frame for the researcher to see once the whale was stationed on the stationing device, indicating that a trial could begin. Whales did not receive feedback or reinforcement for each trial. Each trial required either a response or no response (“go, no-go”), and the response was a conditioned vocal “raspberry” in which the subject let out a sharp sound by exhaling pressurized air through the blowhole. The raspberry was previously trained by use of mimicry and behavioral capturing.

**Figure 2:**
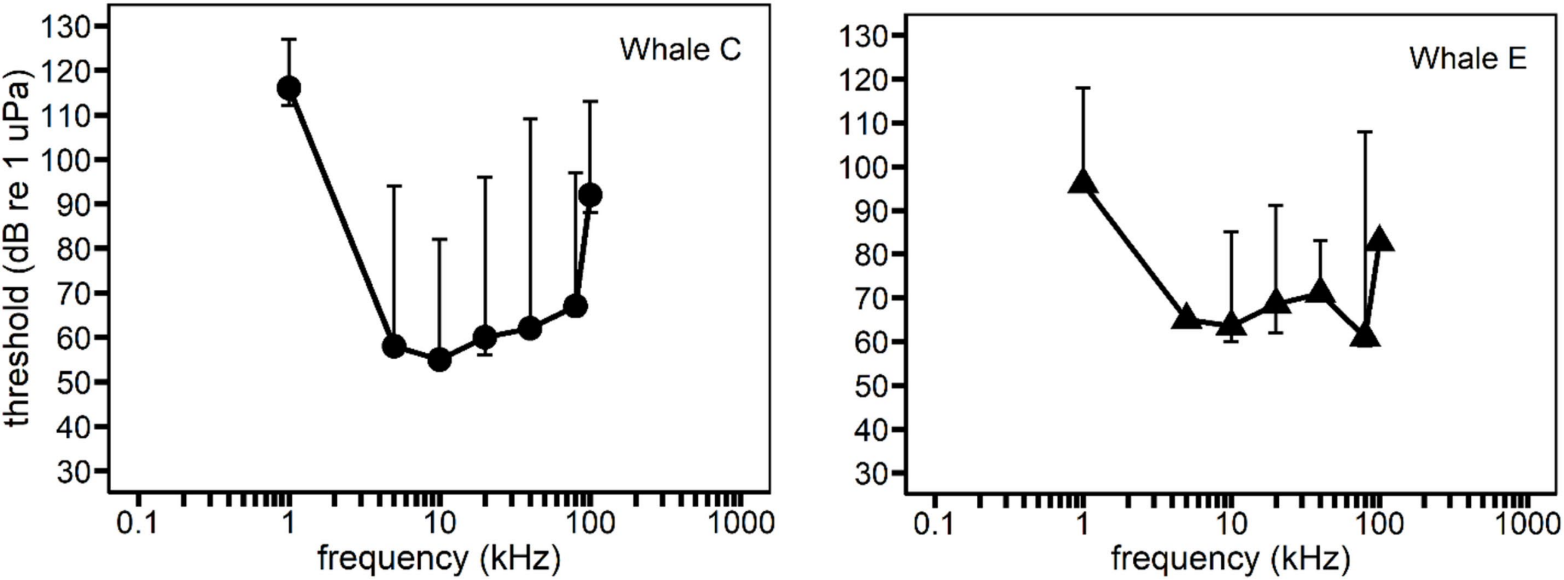
Behavioral audiograms for each whale subject. Solid circles and triangles represent behavioral hearing thresholds for 500 ms duration pure tones. All data was from Branstetter et al. (2023) except for thresholds at 5 and 100 kHz for Whale E (Branstetter et al., 2017). Vertical bars represent the variability in hearing thresholds within a frequency due to the effect of signal duration (see Supplementary Tables S1, S2). Signal duration was accounted for by analyzing sensation level instead of sound pressure level as an independent variable.

An adaptive staircase procedure (Levitt, 1971) was used to determine the progression of sequential trials. The first trial was typically played approximately 20 dB above the expected hearing threshold, and each correct response to a sound signal, or ‘hit’, resulted in a 5 dB decrease for the next signal until the first ‘miss’ (no response to the signal). After the first miss, the signal increased by 2 dB for each miss or decreased by 2 dB for each hit until 6 reversals were obtained (see Figure 2) from Branstetter et al. (2017). A ‘hit’ was logged only if the animal produced a vocal response within a 2 s window following the onset of a tone. Only the vocalizations produced during this trial type were analyzed for response latency. If an animal responded to a ‘catch’ trial, a trial when no signal was played, then a ‘false alarm’ was logged—this response type was not analyzed for response latency.

During most dives, multiple trials were conducted, and the experimenter terminated a dive using a conditioned reinforcer (i.e., “bridge”) only after a correct response, regardless of whether any incorrect responses occurred during the session. The conditioned reinforcer was either a tapping sound made by the trainer, or an underwater sound played from a DAEX24W-8 transducer, (Dayton Audio, Springboro, OH), which instructed the whale to return to the trainer for variable reinforcement. Whales were given various combinations of reinforcement (i.e., food or shaved/cubed ice). Trainers asked for various husbandry behaviors during the sessions to introduce additional opportunities for reinforcement and intermittently confirm the whale’s overall state of motivation and attention during the session. Whales were allowed to terminate any session through non-participation without risk of losing the opportunity to participate in other daily activities or a change in the total amount of reinforcement offered during a day.

### 2.4 Quantitative analysis

#### 2.4.1 Reaction time (RT) measurements

Reaction time was calculated for sounds having frequencies from 1 to 100 kHz and durations from 0.1 ms to 2 s. The HTP software (Finneran, 2003) recorded each trial at a sampling rate of 50 kHz, which was sufficient for capturing the vocal response, and theoretically allowed for a temporal resolution of 20 μs. The HTP software was programmed to record the vocal response of the subject, but was not optimal for recording all of the tonal signals; however, since the tonal signal was always played 500 ms after the start of the trial recording, it was possible to determine reaction time by subtracting 500 ms from the time of vocal response onset.

Reaction time was defined as the duration between the stimulus onset and the onset of the whale’s vocal response and was estimated visually via spectrographic analysis (see Figure 1). The recorded files were imported to Cool Edit Pro (Syntrillium Software, Scottsdale, AZ) for spectrographic analysis of response latency. Only trials with a correct response to the hearing test tone (‘hit’) were used for the analysis. The spectrogram of each. Wav file was viewed to determine where the vocal response began (see Figure 1). The spectrogram was first inspected to ensure that the vocal response was visible and began during the 2 s window of time following the onset of each signal. Any responses that occurred outside of these parameters were not analyzed.

#### 2.4.2 Data analysis

For a single frequency, hearing threshold increases with decreasing signal duration below the auditory integration time. In other words, the auditory integration time is defined as the point at which an increase in signal duration does not affect the hearing threshold (Plomp and Bouman, 1959; Johnson, 1968; Branstetter et al., 2023). Therefore, within a frequency, sound pressure level (SPL) is not comparable across durations below the auditory integration time. Figure 2 illustrates the magnitude of hearing threshold variation within a single frequency due to differences in signal duration. Because hearing threshold data for each sound frequency and duration were available for both subjects (see Figure 2; Supplementary Tables S1, S2), sensation level (SL), defined as the received SPL of the stimulus in decibels above the 50% detection threshold of the subject, was used to account for threshold differences at different signal durations. Auditory integration as a function of frequency for Whale C was described by Branstetter et al. (2023), but threshold data from Whale E were incomplete, and therefore not included in that publication. However, Whale E’s threshold data are suitable for inclusion in the present study because they were used here to derive SLs for reaction times measured during the same hearing tests.

Statistical analysis was performed in RStudio (Posit Software, PBC; version 2023.09.01; ggplot2, stats, moments, and mgcv packages). A generalized linear model (GLM) using a gamma distribution with a logarithmic link function was used to assess the relationship between median RT and frequency and the relationship between RT and sensation level. The model distribution and link functions were determined based on the spread of the data and the Akaike Information Criterion.

Equal latency contours were generated for Whale C using the frequency-specific Pieron Function (Piéron, 1913, 1952),


(1) $$RT=\beta {\left(p^{2}\right)}^{-\alpha }+t0$$

where *ρ* is the intensity of the stimulus (in this case sensation level), α and β are fitting parameters, and t0 is an asymptote corresponding to the minimum amount of time required to respond.

## 3 Results

A total of 225 (145 for Whale C, *n* = 80 for Whale E) hearing test sessions were analyzed with a total of 1,348 (*n* = 838 for Whale C and *n* = 510 for Whale E) individual response latency measurements. Because of the study design and methods, it was not possible to obtain equal sample sizes for all signal conditions (i.e., frequency, SPL and duration combinations), sample sizes were small in some cases (see Table 1), and the data were not normally distributed. Therefore, a non-parametric Mann–Whitney U test was used to assess statistical significance of the effect of the individual whale on overall response latency to determine whether data from both subjects could be combined. Whale C had a median reaction time (RT) of 521 ms, which was significantly faster (z = 5.892, *p* < 0.001) than Whale E which had a median response latency of 658 ms, so analysis of the effect of sensation level, frequency, and duration was conducted separately for each whale. False alarm rates for all sessions ranged from zero to 33%.

**Table 1:** Median reaction time (RT) in milliseconds (ms) and range with sample sizes (*n*) for each frequency for both whales.

	Whale C				Whale E			
Freq (kHz)	*n*	RT (ms)	min	max	*n*	RT (ms)	min	max
1	62	579	403	1,168	113	633	480	1,258
10	115	541	330	1,327	119	675	444	1,489
20	170	530	338	1,487	130	635	449	1,581
40	141	518	369	1,429	27	737	377	1,757
80	221	512	306	1,401	121	706	443	1,542
100	130	477	322	1,392				

**Figure 3:**
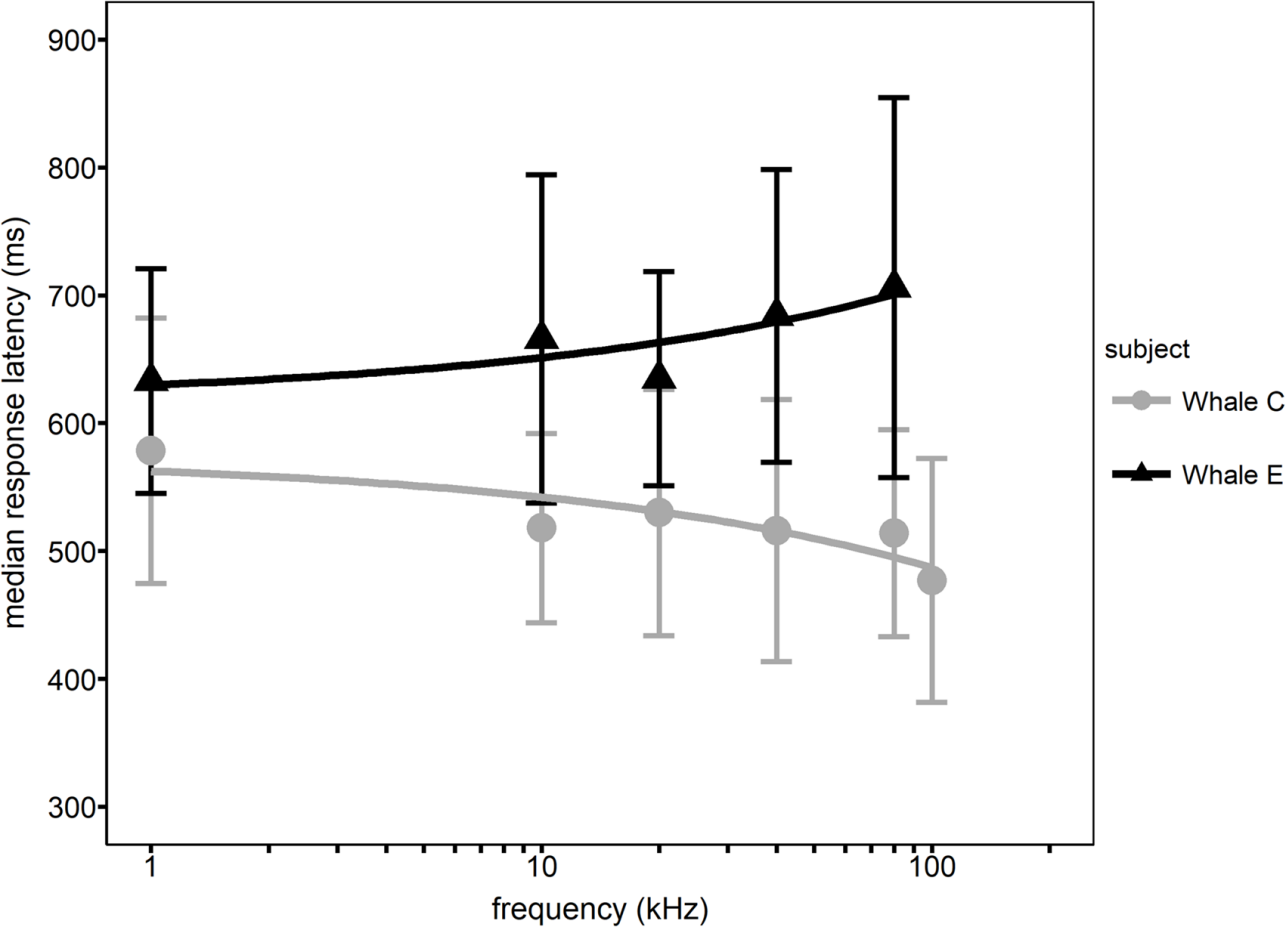
Median response latency (RT) as a function of frequency for each whale. Error bars represent interquartile ranges.

Different RT trends were observed with increasing frequency for both whales. Median RT for Whale C decreased with increasing frequency while median RT for Whale E increased (see Figure 3). A significant relationship between median RT and frequency was observed for Whale C (*F* (1, 4) = 7.748, *p* = 0.0462) but not for Whale E (F (1, 4) = 9.081, *p* = 0.0581).

### 3.1 Response latency as a function of sensation level

**Figure 4:**
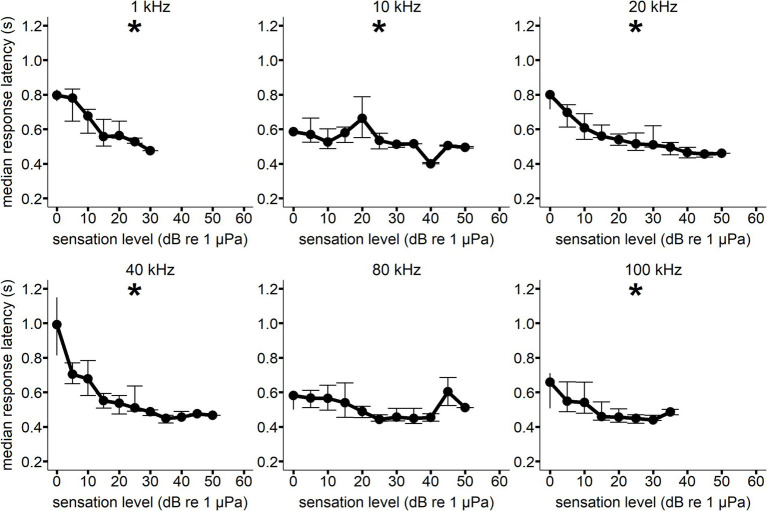
Median response latency as a function of sensation level for Whale C at each hearing test frequency. Error bars show interquartile range. All median response latency values were binned to the nearest 5 dB SL value (i.e., RT values at a SL of 8 dB were included in the 10 dB SL bin). A generalized linear model showed a significant decrease in response latency with increasing sensation level (*p < 0.05) for all frequencies except 80 kHz. Sample size varied between signal conditions.

**Figure 5:**
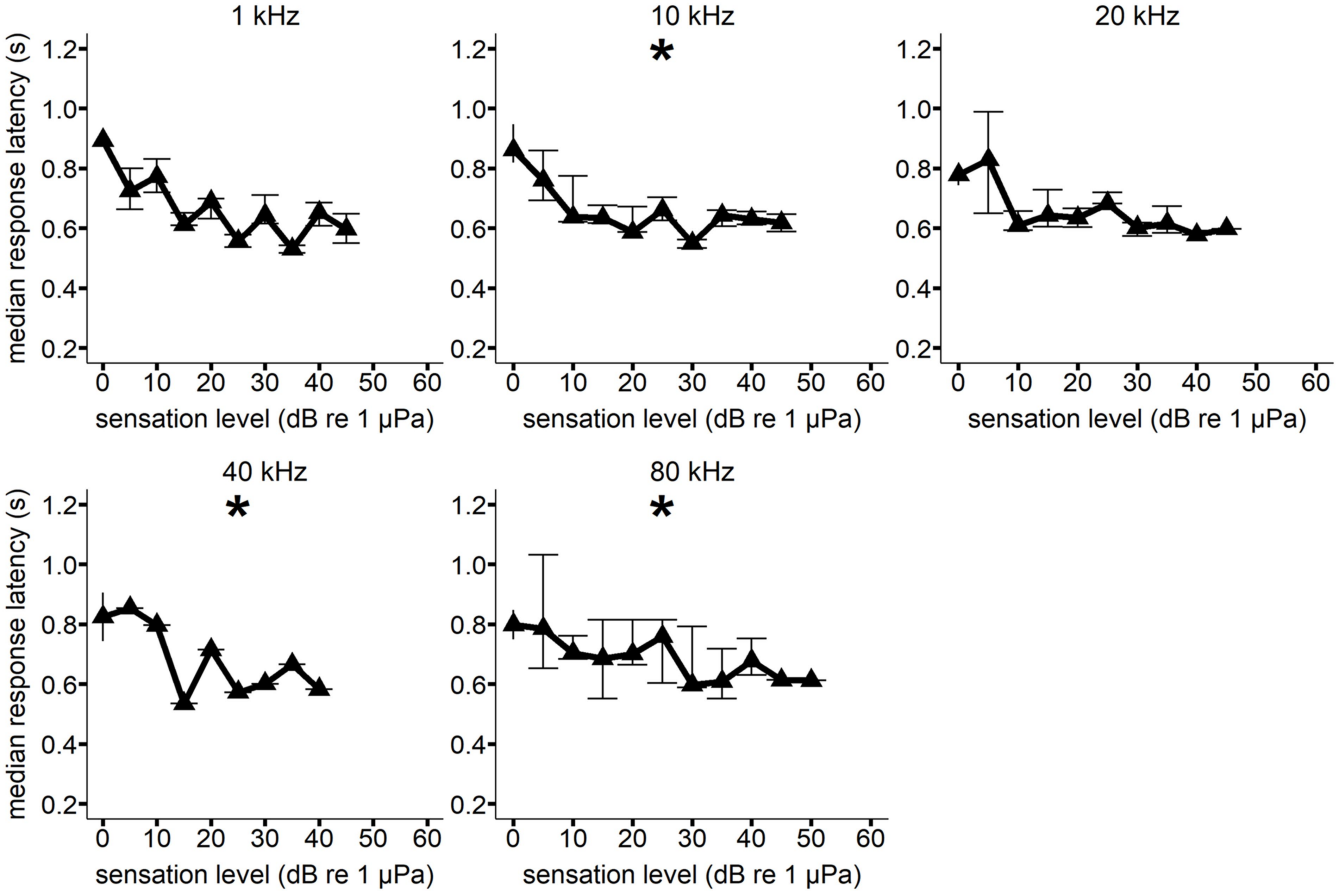
Median response latency as a function of sensation level for Whale E at each hearing test frequency. Error bars show interquartile range. All median response latency values were binned to the nearest 5 dB SL value (i.e., RT values with a SL of 8 dB were part of the 10 dB SL bin). A generalized linear model showed a significant decrease in response latency with increasing sensation level (*p < 0.05) at 10, 40, and 80 kHz. Sample size varied between signal conditions.

A significant decrease in response latency with increasing sensation level was observed for all frequencies but 80 kHz for Whale C, 1 kHz (*F*(1, 5) = 83.644, *p* = <0.001), 10 kHz (*F*(1, 9) = 12.167, *p* = 0.006), 20 kHz (F(1, 9) = 63.991, *p* = <0.001), 40 kHz (F(1, 9) = 30.254, *p* = <0.001), and 100 kHz (*F*(1, 6) = 11.077, *p* = 0.018) (see Figure 4). A significant decrease in response latency with increasing sensation level was observed for all frequencies but 1 and 20 kHz for Whale E, 10 kHz (F(1, 9) = 9.661, *p* = 0.014), 40 kHz (*F*(1, 8) = 8.755, *p* = 0.016), and 80 kHz (*F*(1, 11) = 24.278, *p* = <0.001) (see Figure 5).

**Figure 6:**
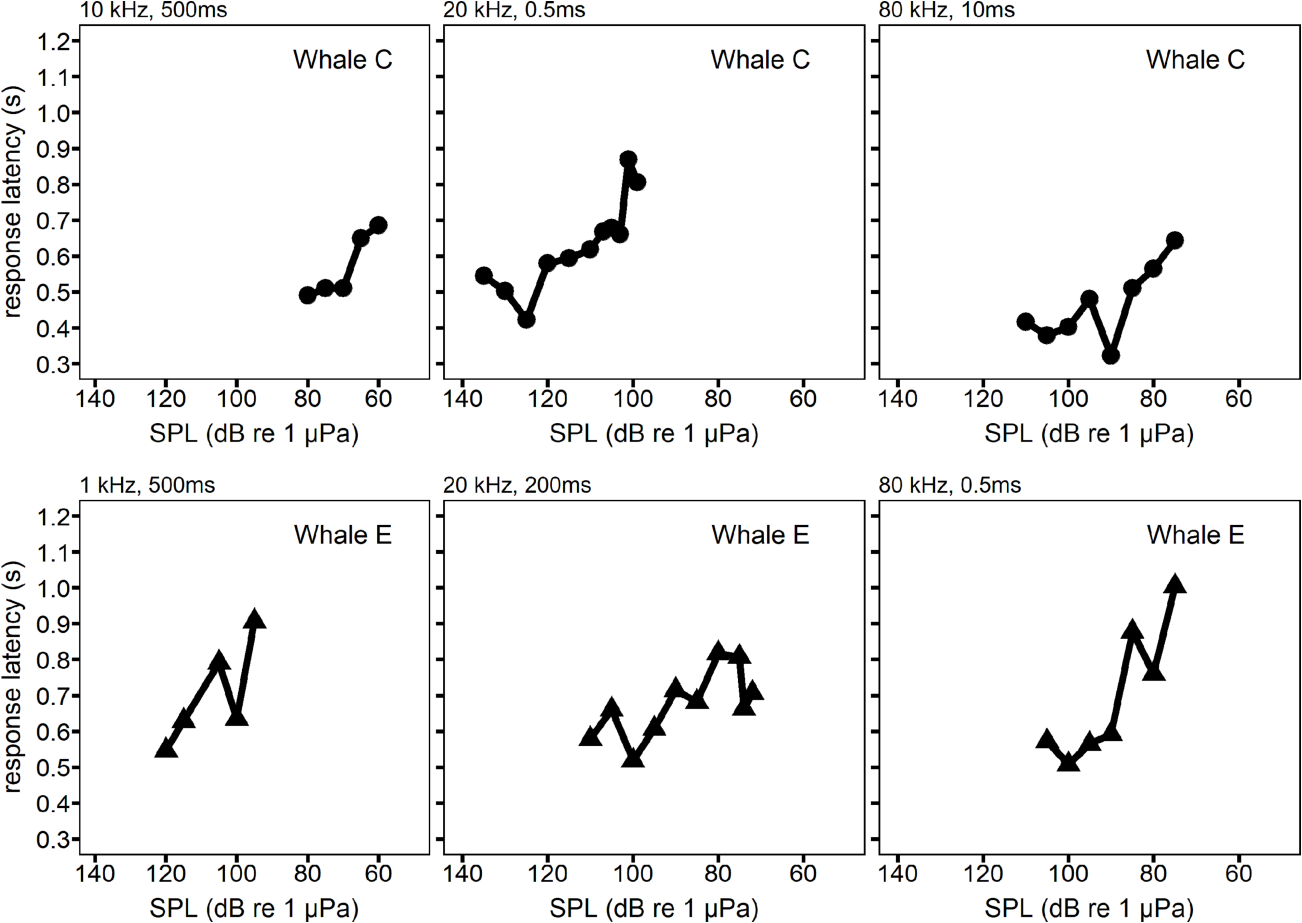
Examples of the change in response latency as a function of sound pressure level (SPL). Each panel is a single hearing test session where the response latency is plotted for sequential trials. SPL decreased over the course of the hearing test, so SPL decreases from left to right in each panel. Whale C is shown in the top row examples. Whale E is shown in the bottom row examples.

An increase in RT with decreasing SPL was also observed during the individual hearing tests. Figure 6 illustrates the progression of individual hearing tests. The x-axis in Figure 6 is reversed to show the higher SPLs first, which matches the progression of sequential trials in each example hearing test.

Generalized linear models indicated no significant relationship between signal duration and median reaction time (RT) for either whale (see Supplementary Figures S1, S2).

### 3.2 Equal latency contours

**Figure 7:**
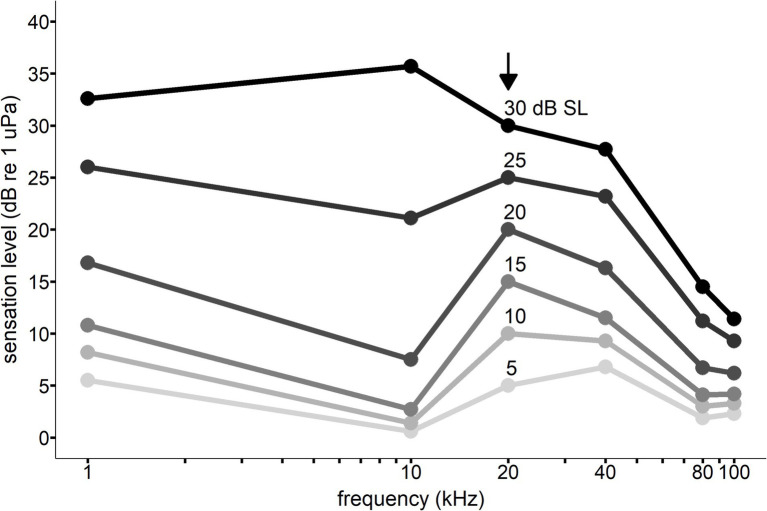
Equal latency contours for Whale C across frequencies. The vertical axis sensation levels are relative to the 20 kHz response latency data indicated by the black arrow. Points along the same line indicate the sensation level at which the response latency is equal to the 20 kHz response latency (resulting from interpolation from the Pieron function described in Equation 1). For example, a 30 dB sensation level signal at 20 kHz would be predicted to have the same response latency as a 36 dB sensation level signal at 10 kHz and a 15 dB sensation level signal at 80 kHz.

The equal latency contours for Whale C were anchored to the 20 kHz data due of the high degree of significance measured between RT and SL and because it falls within the best frequency hearing range of killer whales (see Table 2). The contours were standardized to the 20 kHz RT data at sensation levels 5, 10, 15, 20, 25, and 30 dB. This means that each unique SL/frequency point along a contour line shares the same RT as all other points on the same contour line. For example, a 30 dB SL tone at 20 kHz shares the same RT as a 36 dB SL tone at 10 kHz (see Figure 7).

**Table 2:** Best-fit parameters for the Pieron functions (see Equation 1) used to generate the equal latency contours for Whale C.

	Whale C			
Freq (kHz)	α	β	t0	Adjusted R^2^
1	0.193	0.762	0.300	0.863
10	0.083	0.357	0.300	0.648
20	0.176	0.718	0.300	0.934
40	0.243	0.996	0.300	0.976
80	0.167	0.485	0.300	0.765
100	0.213	0.559	0.300	0.958

The data for Whale E was not suitable to generate equal latency contours.

## 4 Discussion

Here, we measured latencies of conditioned vocal responses in killer whales for the first time. The median RTs at each frequency measured for both whales in the present study ranged from 491 to 705 ms (see Figure 3 in results). In general, other types of non-vocal motor response latencies, such as from a switch release, are more commonly used in equal-latency studies in humans (Wagner et al., 2004; Marshall and Brandt, 1980; Ulrich et al., 1998; Pfingst et al., 1975a,b) and other mammals (Kastelein et al., 2010; May et al., 2009; Green, 1975; Wensveen et al., 2014). Table 3 illustrates the variability of RT measurements among other mammals compared to the present study. The relatively longer RTs in killer whales (see Table 3) are not surprising because of their large size. While nerve conduction occurs at the same maximum velocity, the sensorimotor pathway distance increases with body size (More et al., 2010; More and Donelan, 2018). For the odontocete species in Table 3, RT measurements increase with body size: the harbor porpoise showed a range of non-vocal RTs from 110 to 770 ms (Wensveen et al., 2014), the bottlenose dolphin whistle RTs ranged from 230 to 845 ms (Mulsow et al., 2015), and the killer whale raspberry RTs ranged from 306 to 1,561 ms.

**Table 3:** Studies that observed decreases in reaction time with increasing sensation level or sound pressure level.

Species	Study	Response type	*n*	RT range (ms)	Frequency range (kHz)
Human *Homo sapiens*	Marshall and Brandt (1980)	Non-vocal motor	10	194–290	0.250–4
	Kohfeld et al. (1981)	Non-vocal motor	2	152–323	0.1–10
	Ulrich et al. (1998)	Non-vocal motor	30	170–235	1
	Wagner et al. (2004)	Non-vocal motor	6	182–782	0.940–2
Cat *Felis catus*	May et al. (2009)	Non-vocal motor	6	315–682	0.5–4
Squirrel Monkey *Saimiri sciureus*	Green (1975)	Non-vocal motor	4	245–1,012	0.125–46
Crab-Eating Monkey *Macaca irus*	Stebbins (1966)	Non-vocal motor	2	230–>500	0.250–15
Harbor Seal *Phoca vitulina*	Kastelein et al. (2011)	Non-vocal motor	2	195–990	0.125–100
Harbor Porpoise *Phocoena phocoena*	Wensveen et al. (2014)	Non-vocal motor	1	110–770	0.5–125
Bottlenose Dolphin *Tursiops truncatus*	Mulsow and Finneran (2013) Mulsow et al. (2015)	Vocal	2	230–845	0.125–113
Killer Whale *Orcinus orca*	Present study	Vocal	2	306–1,561	1–100

The column title ‘*n*’ represents the sample size (number of participants) in each study.

The vocal response itself, a trained “raspberry,” likely influenced the reaction times measured in the present study. This is because vocal response evocation—particularly for marine mammals—is probably delayed by the time required to generate sufficient air pressure (Ridgway, 2011; Mulsow et al., 2015). The lowest RT measurement from the present study (306 ms) was longer than the lowest vocal RT measurements in bottlenose dolphins (230 ms) (Mulsow and Finneran, 2013; Mulsow et al., 2015). This could be due to the considerable size difference between the two species, or the type of vocal response (e.g., whistles). Although the type of response measured in the present study is different from previous work in odontocetes, the observed decrease in median RT with increasing sensation level in the present study (see Figures 4, 5) is consistent with the previous research in bottlenose dolphins (Mulsow et al., 2015; Finneran and Schlundt, 2011; Mulsow and Finneran, 2013) and the harbor porpoise (Wensveen et al., 2014).

The individual session data from the present study demonstrated that an increase in RT occurred as SPL decreased within a hearing test session (see Figure 6). Reaction time for both vocal responses and non-vocal motor responses has been shown to decrease with increasing SPL until it plateaus (see Table 3). The present study supports these findings at most of the frequencies measured with both whales. The gradient of the median RT curves depicted in Figures 4, 5 exhibited a noticeable reduction in steepness within the 10–20 dB range for several of the measured frequencies. A similar trend was observed in bottlenose dolphins (Mulsow et al., 2015). The RT curves in the present study were not smooth at all frequencies. For example, Whale C had a higher median RT than expected at 20 dB SL for 10 kHz and 45 dB SL for 80 kHz. Whale E had a lower median RT than expected at 15 dB SL for 40 kHz. The unequal sample sizes among the SLs at each of the frequencies for both whales likely contributed to this variation in median RT.

The two whale subjects’ RTs differed, with Whale C having substantially lower RTs than Whale E (see Table 1), on the order of approximately 100 ms. Ridgway (2011) observed similar magnitude (100–150 ms) between-subject differences in bottlenose dolphin vocal RTs, with the youngest subject having the lowest RTs. It is unlikely that age, body size or other physical differences between the two whales were the source of this individual variation, as the whales were similar in size and age (see Section 2.1). For both subjects, response latencies for long-duration signals (> 500 ms) often resulted in the vocal response overlapping with the test signal. In other words, neither whale waited for the tone to end before responding. One possible explanation for the observed subject-specific differences in RT was Whale C’s additional experience with behavioral hearing tests compared to Whale E. While both whales participated in tone detection (Branstetter et al., 2017) and auditory masking studies (Branstetter et al., 2021, 2024), Whale C began his initial training several months before Whale E and Whale C completed data collection for a temporal integration study where Whale E did not (Branstetter et al., 2023). Interestingly, the two subjects showed diverging trends in RT as a function of sound frequency (see Figure 3). Whale E showed an increase in RT with increasing frequency, and Whale C showed a decrease in RT with increasing frequency for all sensation levels. Data from Branstetter et al. (2023) does not demonstrate hearing loss at 80 kHz for Whale E (see Supplementary Table S2), suggesting that other factors may influence the difference between the two subjects’ relationship between sound frequency and RT.

The RT dataset had a low sample size at many of the short duration signals (< 20 ms) making it difficult to differentiate an effect on RT from signal duration (see Supplementary Figures S1, S2; Figure 2). Previous studies have analyzed the relationship between RT and signal duration in humans. Ulrich et al. (1998) observed a decrease in RT with increasing signal duration to a specific point before RT plateaued, which matches the relationship between hearing threshold and signal duration in Branstetter et al. (2023). Therefore, the effect of signal duration on RT was likely captured by using SL in the present study.

The equal-latency contours in the present study collapsed closer together at higher frequencies, indicating that sensation level affected response latency less than at lower frequencies (also see Figures 4, 5). Compression of the equal latency contours at the upper and lower frequencies has been observed in previous studies (Mulsow et al., 2015; Wensveen et al., 2014; Kastelein et al., 2011; May et al., 2009; Marshall and Brandt, 1980). It was surprising that compression was not observed at lower frequencies (1 kHz) where the hearing thresholds were highest for Whale C, but sample size was lowest for this frequency (see Table 1), which may have influenced this result. However, killer Whale communication calls often contain peak energy at lower frequencies (1–3.5 kHz) (Williams et al., 2014a), and this biological relevance could affect loudness perception. The contours were expected to flatten out at higher sensation levels, as higher intensity signals have been shown to reduce the effect of frequency on equal loudness and equal latency contours (Kohfeld et al., 1981). This phenomenon is likely dependent on neural rise time rates at the onset of the signal, which reach a maximum at higher intensities (Kohfeld et al., 1981). While the 25 and 30 dB sensation level contours did exhibit some flattening across the lower frequencies, the higher frequencies showed lower equal latency SLs than expected based on previous research.

At 80 and 100 kHz, the results of the equal latency contours for Whale C (see Figure 7) do not conform to the hypothesized audiogram-like shape. The high-frequency portion of the equal latency contours was compressed at lower sensation levels compared to frequencies within the best hearing range (5–20 kHz) for Whale C (see Figure 2). Equal latency contours from previous studies with marine mammals have shown the contours to follow similar trends as the behavioral audiogram, with lower levels required to elicit the same RTs at more sensitive hearing frequencies compared to frequencies outside the best range of hearing (Mulsow et al., 2015; Finneran and Schlundt, 2011). Overall, the equal latency contour data in the present study do not consistently support that lower SLs are required to elicit equal RTs in the best hearing range, or that equal-latency contours follow similar trends as the audiogram. The gradual onset of the raspberry vocal response and methods used in the present study likely introduced variability that influenced the equal latency contours. Further research on response latency in odontocetes would benefit from using a vocal response with a rapid onset (e.g., sonar clicks or burst pulse) or a non-vocal motor response—such that automated detection methods such as pressure or light sensors—could reduce response onset uncertainty.

Overall, the relationship between response latency and sensation level observed in the present study supports that RT can be used as a proxy for perceived loudness growth in killer whales. These first measurements of killer whale vocal response latencies provide insight into the relationship between auditory sensitivity and loudness perception in large odontocetes. Further study of loudness perception is important for understanding the vulnerability of these animals to acoustic disturbance, and for informing conservation efforts.

## Data Availability

The raw data supporting the conclusions of this article will be made available by the authors, without undue reservation.
