# Supplementary material for: Latencies of conditioned vocal responses to hearing test tones in killer whales (Orcinus orca)
Source: Front Behav Neurosci. 2025 Jan 29;18:1495579. doi: 10.3389/fnbeh.2024.1495579 (PMC11836954; doi:10.3389/fnbeh.2024.1495579)
Supplement: Supplementary file 1 [file Data_Sheet_1.PDF]

Hearing Thresholds

Table S1. Duration and frequency-specific average thresholds (dB) for Whale C (previously published by Branstetter et al. 2023).

	Signal duration (ms)												
Frequency (kHz)	0.05	0.1	0.2	0.5	1	5	10	50	100	200	500	1000	2000
1						127	129	119	116	116	116	111	112
10				82	76	66	67	59	60	55	55	54	
20				96	87	71	65	63	65	63	60	56	64
40				109	100	81	85	67	66	64	62	60	60
80				97	91	83	74	75	65	67	67	69	73
100	113	107	99	97	101	88	89	88	92	93	92	88	91

Table S2. Duration and frequency-specific average thresholds (dB) for Whale E from the same experiment conducted by Branstetter et al. (2023).

	Signal Duration (ms)										
Frequency (kHz)	0.2	0.5	1	5	10	50	100	200	500	1000	2000
1				118	115	104	97	96	96	97	95
10		85	80	75	71	67	65	66	64	60	
20		88	91	86	84	68	71	64	69	62	67
40					83		72		71		
80	108	94	92	71	72	71	62	59	61		

Duration Analysis

Statistical analysis indicated that reaction time (RT) did not depend on sound duration within a frequency for near-threshold (<15 dB sensation level) or supra-threshold (>15 dB sensation level) signals (see figure S1 and S2). Significant variation between supra-threshold and near-threshold signals across duration was observed at 20 kHz, 40 kHz, and 80 kHz for Whale C and only at 10 kHz for Whale E (Mann Whitney U Test, $p < 0.05$).

The results of this study did not show a clear relationship between signal duration and reaction time (see Figure S1 and S2; Figure 2). The dataset used in this study provided a low sample size at many of the short duration signals (<20ms) making it difficult to differentiate an effect on RT from signal duration. Previous studies have analyzed the relationship between RT and signal duration in humans. Ulrich et al. (1998) observed a decrease in RT with increasing signal duration to a specific point before RT plateaued, which matches the relationship measured between auditory threshold and signal duration in Branstetter et al. (2023). Since RT was shown to decrease with increasing sensation level in this study, further research should be conducted to analyze the relationship between signal duration and reaction time.

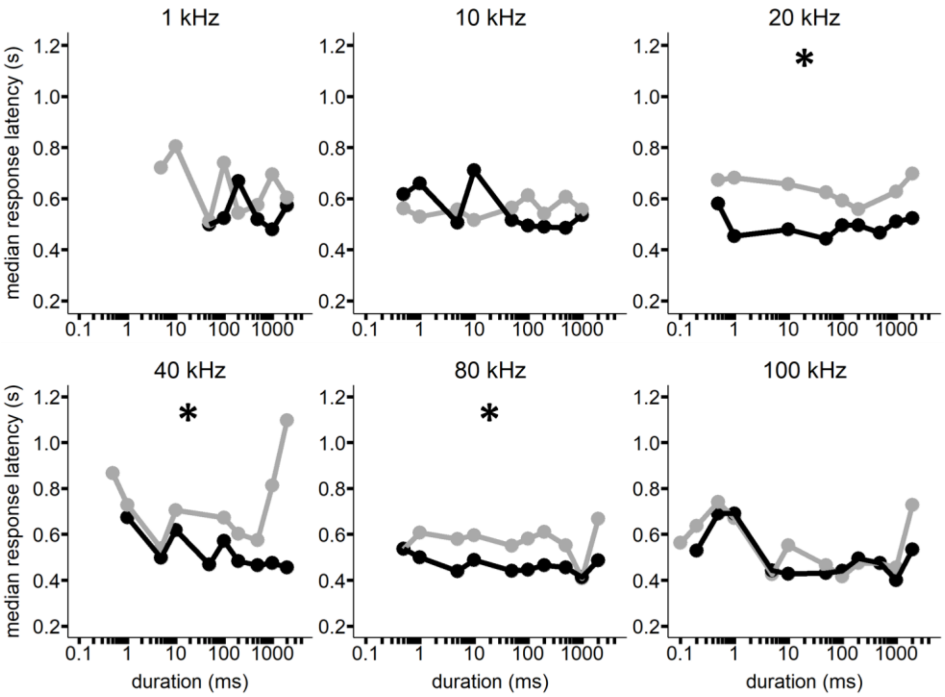

Figure S1: Median response latency (RT) as a function of signal duration for Whale C. Near-threshold (NT) sensation levels < 15 dB above threshold are shown in gray and supra-threshold (ST) sensation levels > 15 dB above threshold are shown in black. A significant difference between ST and NT response latencies was found for 20, 40, and 80 kHz (Mann Whitney U Test, * = $p < 0.05$). No significant relationship between median response latency and signal duration was measured.

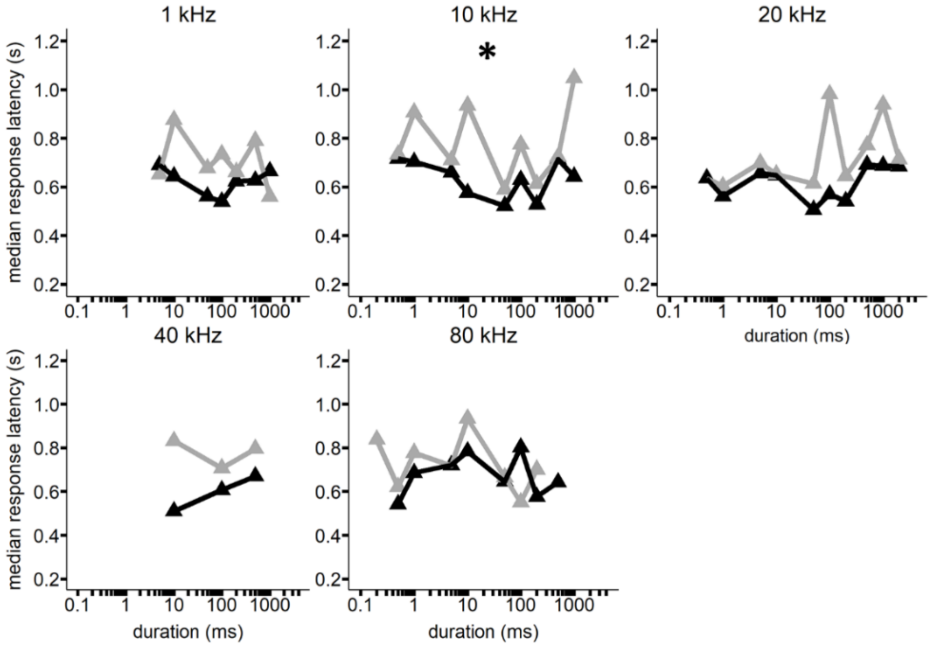

Figure S2: Median response latency (RL) as a function of signal duration for Whale E. Near-threshold (NT) sensation levels < 15 dB above threshold are shown in gray and supra-threshold (ST) sensation levels > 15 dB above threshold are shown in black. A significant difference between ST and NT response latencies was found for 10 kHz (Mann Whitney U Test, * = $p < 0.05$). No significant relationship between median response latency and signal duration was measured.

References

Ulrich, R., Rinkenauer, G., & Miller, J. (1998). Effects of stimulus duration and intensity on simple reaction time and response force. *Journal of Experimental Psychology: Human Perception and Performance*, *24*(3), 915.
